# Case Report: Successful Management of a 29-Day-Old Infant With Severe Hyperlipidemia From a Novel Homozygous Variant of *GPIHBP1* Gene

**DOI:** 10.3389/fped.2022.792574

**Published:** 2022-03-10

**Authors:** Shu Liu, Zhiqing Wang, Xianhua Zheng, Ye Zhang, Sisi Wei, Haimei OuYang, Jinqun Liang, Nuan Chen, Weihong Zeng, Jianhui Jiang

**Affiliations:** ^1^Children Inherited Metabolism and Endocrine Department, Guangdong Women and Children Hospital, Guangzhou, China; ^2^Guangdong Provincial Key Laboratory of Gastroenterology, Department of Gastroenterology, Nanfang Hospital, Southern Medical University, Guangzhou, China; ^3^Department of Clinical Laboratory, Guangdong Women and Children Hospital, Guangzhou, China

**Keywords:** severe hyperlipidemia, whole exome sequencing, *GPIHBP1* gene, novel variant, therapy

## Abstract

**Background:**

Severe hyperlipidemia is characterized by markedly elevated blood triglyceride levels and severe early-onset cardiovascular diseases, pancreatitis, pancreatic necrosis or persistent multiple organ failure if left untreated. It is a rare autosomal recessive metabolic disorder originated from the variants of lipoprotein lipase gene, and previous studies have demonstrated that most cases with severe hyperlipidemia are closely related to the variants of some key genes for lipolysis, such as *LPL, APOC2, APOA5, LMF1*, and *GPIHBP1*. Meanwhile, other unidentified causes also exist and are equally worthy of attention.

**Methods:**

The 29-day-old infant was diagnosed with severe hyperlipidemia, registering a plasma triglyceride level as high as 25.46 mmol/L. Whole exome sequencing was conducted to explore the possible pathogenic gene variants for this patient.

**Results:**

The infant was put on a low-fat diet combined with pharmacological therapy, which was successful in restraining the level of serum triglyceride and total cholesterol to a low to medium range during the follow-ups. The patient was found to be a rare novel homozygous duplication variant-c.45_48dupGCGG (Pro17Alafs^*^22) in *GPIHBP1* gene-leading to a frameshift which failed to form the canonical termination codon TGA. The mutant messenger RNA should presumably produce a peptide consisting of 16 amino acids at the N-terminus, with 21 novel amino acids on the heels of the wild-type protein.

**Conclusions:**

Our study expands on the spectrum of *GPIHBP1* variants and contributes to a more comprehensive understanding of the genetic diagnosis, genetic counseling, and multimodality therapy of families with severe hyperlipidemia. Our experience gained in this study is also contributory to a deeper insight into severe hyperlipidemia and highlights the importance of molecular genetic tests.

## Introduction

Severe hyperlipidemia, with a typical feature of high concentration of lipidemia in fasting state, is primarily attributed to a genetic defect in intravascular lipolysis. Urgent clinical intervention is always required, especially for serious cases in that some severe complications, otherwise, may follow, such as pancreatitis, which contributes to 5–6% of the overall mortality, as well as pancreatic necrosis or persistent multiple organ failure that have been proved to be associated with the highest mortality rate (1). Amongst all the subtypes of severe hyperlipidemia, monogenic forms are the ones linked to the defects in metabolism of triglyceride (TG)-rich lipoproteins, namely lipolytic cascade. These defects may be caused by variants in no less than five different genes, predominantly (about 95%) inherited ones in both alleles of the lipoprotein lipase gene, which, aliased as *LPL* (2), is responsible for encoding of the enzyme lipoprotein lipase (LPL; OMIM #238600). Previous studies have shown that the defects directly related to *LPL* gene variants are also relevant to the majority of severe hyperlipidemia cases (3–6). The rest 5% are the results of variants in other genes involving in LPL functioning, including *APOC2* (encoding apolipoprotein CII, activator of LPL; OMIM #207750) (7, 8), *APOA5* (encoding apolipoprotein AV, activator of LPL; OMIM #144650) (9, 10), *LMF1* (encoding lipase maturation factor 1, a tissue factor triggering the secretion of functional LPL and hepatic lipase; OMIM #611761) (11, 12), and *GPIHBP1* (encoding glycosylphosphatidylinositol-anchored highdensity lipoprotein-binding protein 1, the molecular platform by which LPL is able to interact with TG-rich lipoproteins, apolipoprotein CII, and apolipoprotein AV on the endothelial surface of capillaries; OMIM #612757) (13, 14). Meanwhile, there are also other variants to be identified (3), and all these variants may result in LPL malfunction.

GPIHBP1, categorized as one of the lymphocyte antigen 6 (Ly6) family, is a capillary endothelial cell protein consisting of 184 aa. Being a critical protein for LPL transportation from the subendothelial spaces to capillary lumen, GPIHBP1 can promote lipolysis by working as the main site to bind LPL on endothelial surface (15). So, variants of *GPIHBP1* may disturb lipolysis process and thus result in severe hyperlipidemia. However, although previous studies so far have offered some leads to the lipolytic mechanism of GPIHBP1 and disclosed a handful of case reports concerning *GPIHBP1* variants, our understanding on *GPIHBP1*-deficient diseases is still to be deepened. In this study, we described the clinical features, genetic analysis and our hands-on experience of successful pharmacological management of a 29-day-old baby with abnormally elevated plasma TG (25.46 mmol/L), who was subsequently identified as a rare novel homozygous duplication variant in *GPIHBP1* gene as the cause of her severe hyperlipidemia.

## Materials and Methods

### Plasma Lipid Profile Analysis

Blood samples were collected after fasting for 6 h. The levels of serum glucose (Glu), TG, TC, HDL, LDL, APO-A1, and APO-B were measured enzymatically using an automatic analyzer (ADVIA 2400, SIEMENS, Germany).

### Genetic Analysis

Enlightened by the abnormal clinical findings, hereditary hyperlipidemia was taken into the pediatricians' consideration for further confirmation using whole exome sequencing (WES, Illumina, San Diego, CA, USA). Details of the experiments, mutation frequency investigation, variant verification, and assessment of the conservation of amino acid residues were displayed in the Supplementary Material (shown in Supplementary Material-genetic analysis). The pathogenicity of the sequence variants was interpreted according to the American College of Medical Genetics and Genomics/Association for Molecular Pathology (ACMG/AMP) guidelines (16, 17).

The study was approved by the Ethics Committee of Guangdong Women and Children Hospital. Written informed consent was obtained from the patient's legal guardian for the publication of any potentially identifiable images or data included in this article. All the procedures carried out in our study were strictly in accordance with the Declaration of Helsinki.

## Retrospective Analysis of Patient's Medical Records Concerning Demographics, Clinical Signs and Symptoms, Results of Biochemical, and Genetic Analysis

### Clinical Information

The patient was a 29-day-old Chinese female infant with a chief complaint of “irritability, fever, vomiting and convulsion for 2 days,” who was enrolled in our study after consultation in the pediatric clinic of our hospital. The patient responded poorly to the treatment in previous visits to her local hospitals due to the limited health care resources there, making her condition worsening with the time. Therefore, she was referred to our hospital and finally placed in the pediatric intensive care unit. The infant is the first child of the non-consanguineous parents, with a full-term birth weight of 2,600 g. After admission to hospital, a physical examination was performed in the wake of history collection. The height of this exclusively breastfed infant was 52 cm (22.6th percentile), and the weight 3.7 kg (16.9th percentile). Appearance of low weight, malnutrition, poor response to external stimuli, and somnolence were noticed. Movement range of the joints and muscular tension of the four limbs were normal. Abdominal palpation indicated normal liver and spleen volume. After aggressive treatment, the infant was stabilized in vital signs, with physical examinations showing normal body temperature (axillary temperature, 37.1 degrees Celsius), normal muscle strength, muscle tension and physiological reflexes.

Unexpected discovery arose from milky blood sample (lactescent plasma) and the results of lipid testing (in fasting state), which revealed abnormally elevated TG (25.46 mmol/L; normal range, 0.46 to 2.28 mmol/L) as well as abnormal cholesterol metabolism-total cholesterol (TC, 14.53 mmol/L; normal range, 3.12 to 5.68 mmol/L), high-density lipoprotein cholesterol (HDL-C, 1.12 mmol/L; normal range, 0.91 to 2.18 mmol/L), and low-density lipoprotein cholesterol (LDL-C, 2.01 mmol/L; normal range, 1.28 to 3.41 mmol/L). The first therapeutic intervention for the infant was oral therapy free of long chain fatty acids, supplemented with high protein, high carbohydrate, and medium chain triglycerides (MCT), which lasted for 5 days but failed to control hyperlipidemia. Due to the potential risk of acute pancreatitis associated with high levels of plasma TG and the signs of hypoproteinemia as demonstrated by her low weight as well as a total serum protein and albumen of 51.2 and 28.8 g/L, respectively, enteral feeding was discontinued at the day when the baby was 1 month and 5 days old, and substituted by multimodality therapy including fasting, nasogastric drainage, total parenteral nutrition (TPN), and anti-infection treatment. After 7 days (1 month and 12 days old), plasma TG level decreased significantly to 1.50 mmol/L and TC to 9.66 mmol/L. Whereas, an evident rise in plasma TG up to 3.77 mmol/L was, again, detected after switching to breastfeeding for 5 days (1 month and 17 days old). So the infant was put on a low-lipid formula diet, mainly STOLLE milk powder (Stolle Milk Biologics International Incorporated, New Zealand), a kind of formula low in fat and high in protein with proper amount of water-soluble vitamins added (the details of dietary interventions was shown in Supplementary Table 1). As a result, the plasma TG level declined promptly, ending up at 2.92 mmol/ L when the infant was discharged from the hospital (2 month and 3 days old).

**Figure 1:**
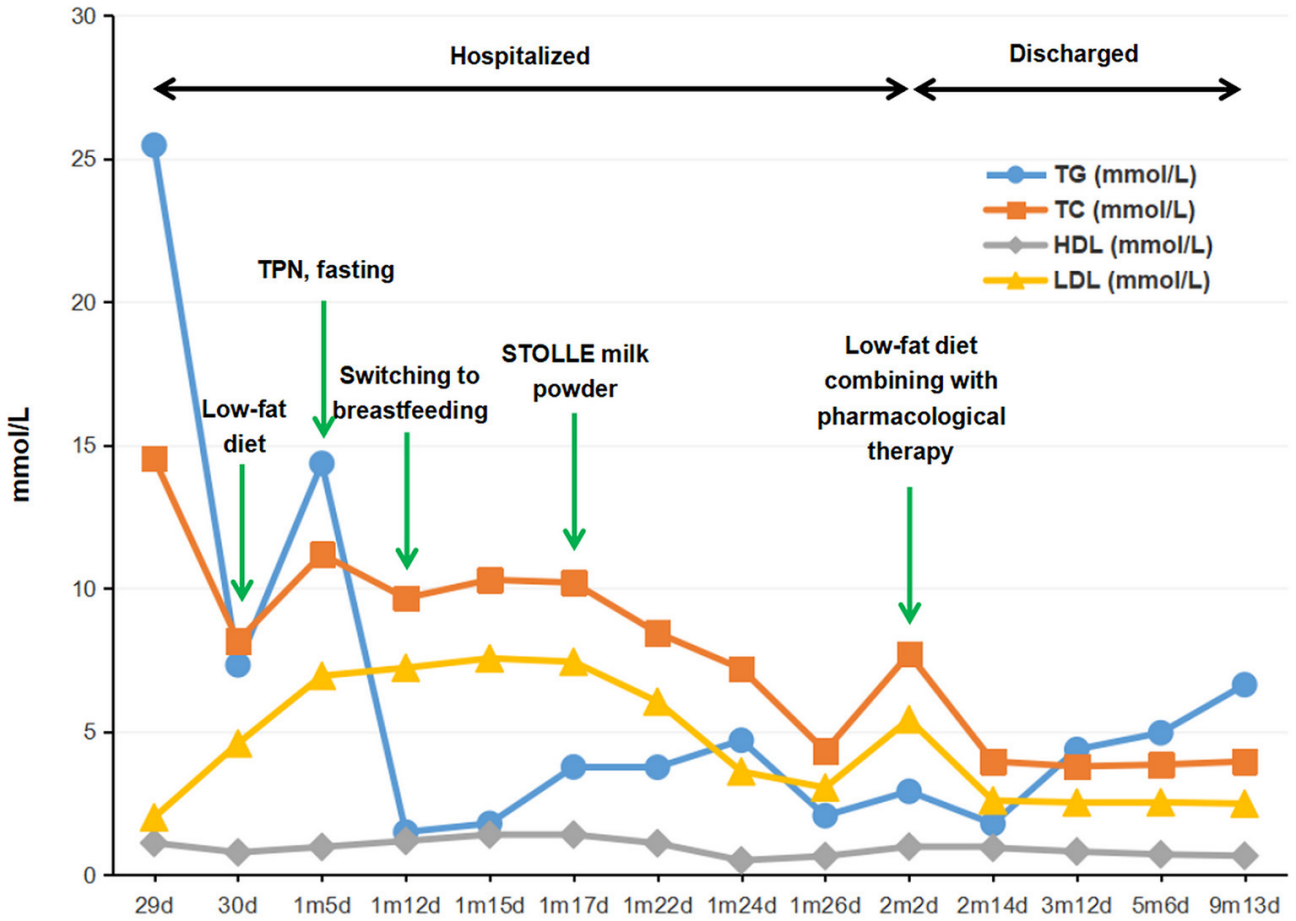
Flow-chart illustrating the management procedure and the serum levels of TG, TC, HDL-C, and LDL-C of the proband. TG, triglyceride; TC, total cholesterol; HDL-C, high-density lipoprotein cholesterol; LDL-C, low-density lipoprotein cholesterol; TPN, total parenteral nutrition.

Considering the evidence of lipometabolic disturbance and presumed diagnosis of severe hyperlipidemia, the infant, after discharged, continued to be kept on a low-fat and high-carbohydrate protocol with proper amount of protein diet and supplementation of L-carnitine, coenzyme Q10 as well as water-soluble vitamins (B1, B2, B6, B12, C, and folic acid) during her treatment at home. Individualized dosage was given for her medication protocol and adjusted accordingly based on her clinical manifestation, the level of blood lipid, and other nutritional indicators (shown in Supplementary Table 2). Surveillance of her medical condition was also performed to avoid potential complications. Meanwhile, the infant continued to take examinations in our outpatient clinic, irregularly, on and off, because of poor compliance. In the follow-ups, the baby was monitored by serum TG, TC, HDL-C, LDL-C, apoprotein A1 (APO-A1), apoprotein B (APO-B), and glucose every 3 to 6 months (shown in Supplementary Table 3). Besides, evaluation of her physical development was conducted at a 3 month interval. The results revealed that the serum TG and TC kept fluctuating from low to medium levels (Figure 1), and in the last follow-up when the baby was 9 months old, she was 8.6 kg (42.2th centile) in weight and 72 cm (56.1th centile) in height, with a head circumference of 44.5 cm (56.9th centile), indicating a good health and normal development. Likewise, laboratory tests also showed dramatic improvement as compared with her first visit, reflecting a sound developmental condition of the patient as well as the beneficial outcomes of this treatment protocol.

Based on the above findings and the latest research consensus (18), the baby was suspected of severe hereditary hyperlipidemia (triglyceride concentration >10 mmol/L), which is likely to have a monogenic cause, despite the fact that the patient's parents were healthy with normal levels of serum lipid and lipoprotein, and that the family history indicated neither hyperlipidemia nor pancreatitis. Therefore, aiming to identify the possible pathogenic gene variants for this infant, we performed this study using genetic analysis tools, with 100 Chinese healthy subjects recruited as the control group.

### Genetic Findings

**Figure 2:**
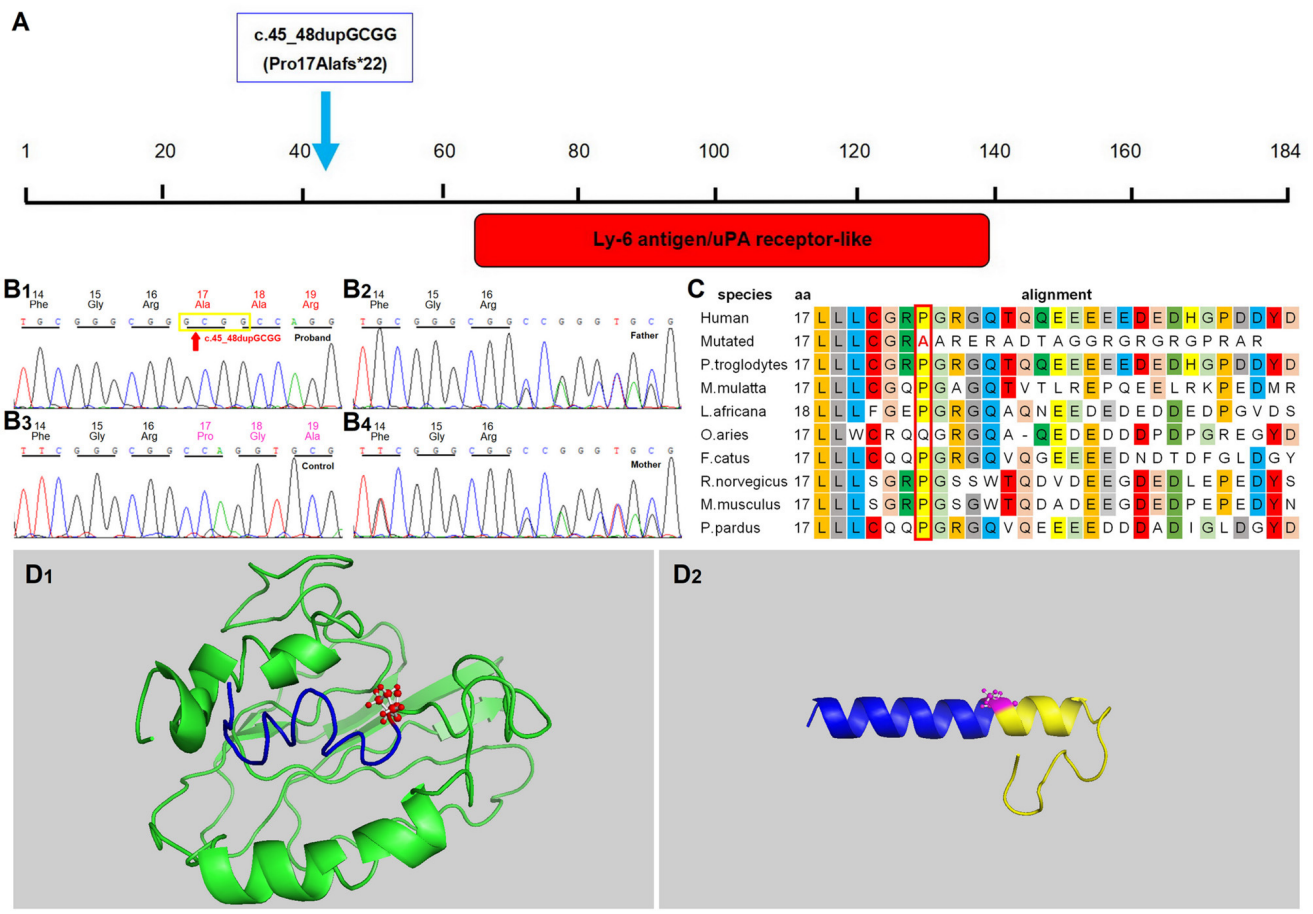
Identification of the variant in *GPIHBP*1 gene. **(A)** Graphical view of the protein domain and structure of *GPIHBP*1. The red box stands for the Ly-6 antigen/uPA receptor-like domain (IPR016054). The homozygous variant c.45_48dupGCGG (p.Pro17Alafs*22) is marked by wathet blue arrow. **(B)** Sequence chromatograms of the *GPIHBP*1 variant. **(B1–B4)** indicates the variant sequences of c.45_48dupGCGG in the proband, his father, mother and normal controls, respectively. The duplication variant site is indicated by red arrow. **(C)** BLAST comparison of the sequence around amino acids 17 in orthologs of GPIHBP1 among various species. The block and colored characters represent highly conserved amino acids among these species, while the white shading stands for inconsistent residues. **(D)** Structural difference between wild-type and C-terminal frameshift GPIHBP1 proteins. Efforts were made to predict the functions of the mutant protein and the wild-type (full-length) GPIHBP1. The full length wild-type protein is exhibited in the left panel **(D1)**, and the mutant protein (p.Pro17Alafs*22) in the right panel **(D2)**. As shown in the mutant GPIHBP1 proteins **(D2)** model, the frameshift originated from p.Pro17Alafs*22 variant is highlighted in yellow, corresponding to the wild-type portion depicted in green **(D1)**. Meanwhile, the consistent part of the N-terminus of the wild-type and the mutant protein is show in blue. It can be seen that the variant compromises the original structure and integrity of the GPIHBP1 protein.

During hospitalization, the WES for this infant revealed the presence of a homozygous duplication variant-c.45_48dupGCGG (Pro17Alafs^*^22)-in exons 1 of *GPIHBP1* gene (Figures 2A,B). However, being heterozygote carriers of the variant, the parents did not show any symptoms of the disorder. This variant was computationally predicted to be deleterious and morbigenous by CADD, PROVEAN, and Mutation Taster. Results of bioinformatic analysis also strongly suggested it a disease-causing variant. Moreover, this variant was not detected in any of the 100 Chinese healthy subjects in the control group, and also had not been disclosed by any public databases as mentioned above, thus ruling out the possibility of a polymorphism and suggesting its novel and rare occurrence. Ultimately, the variant was identified as a pathogenic factor according to the Sherloc/ACMG criteria: it was not recorded in any of the existing population databases, and was assumed to be closely associated with a highly conserved amino acid, and also, to be disease-causing based on *in silico* algorithms used for pathogenicity prediction (17). In this case, the c.45_48dupGCGG duplication variant in exon 1 led to the substitution of the remaining 168 C-terminal amino acid residues with 21 mutant ones (Figure 2D), and the Ly-6 antigen/uPA receptor-like domain of this protein was also affected, thus likely to cause a direct impairment on the enzymatic activity of GPIHBP1. Meanwhile, the functional significance of the mutated amino acid is evident considering its high evolutionary conservation from mammals to invertebrates (Figure 2C). The outcomes from bioinformatic analysis strongly support that the novel homozygous variant c.45_48dupGCGG(Pro17Alafs^*^22) is with a disease-causing nature.

As far as we know, this is, in Chinese population, the first report of baby patient with hyperlipidemia due to *GPIHBP1* variant, and also the first case with the variant of c.45_48dupGCGG(Pro17Alafs^*^22) in *GPIHBP1* gene that was disclosed worldwide among patients with severe hyperlipidemia.

## Discussion

### Clinical Management

In the present study, we delineated a 29-day-old female baby with incidentally found severe hereditary hyperlipidemia originated from a novel and homozygous *GPIHBP*1 duplication variant. During the treatment, the elevated blood lipid associated with a possible risk of pancreatitis was promptly reduced to a safe level, which, we believe, was attributed to the early detection of the abnormalities in this patient, as well as the timely and effectively clinical intervention. The further administrated low-fat diet combined with pharmacological therapy, a success in constraining the blood lipid within a lower range, was considered a key contributor to the normal and healthy development of the baby over time. As things stand, hereditary hyperlipidemia, being a kind of ultra-rare monogenic disease, was commonly spotted by blood serum screening among the suspects-adults and children with obesity, pancreatitis, or xanthoma. However, even the reported cases of little baby or infant with hereditary hyperlipidemia, which were actually very few, were always with a chief complaint of fever (19, 20), vomiting (21), irritability (20), hemoptysis (22), or jaundice (23) instead of something directly linking to a confirmed diagnosis of hyperlipidemia. Therefore, it was not uncommon that the disease, in some cases, was not even on the suspect list of the pediatricians in the first place. All these facts remind us that we should be more alert and sensitive to the clues (such as the results of lipid tests) hidden behind the common presentations of infancy hyperlipidemia-a disease worthy of higher attention and deeper research. Only in this way can proper diagnostic tools (such as genetic analysis) possibly be recruited for further identification. In this study, our findings from genetic analysis have revealed the key role of genetic defects as a predisposing factor of severe hereditary hyperlipidemia, which also sheds a new light on the complexity underlying the etiology of these phenotypes.

It has been known that there are various ways to treat hyperlipidemia, including low-fat diet, pharmacological therapy, and even plasma exchange. Which one to choose usually depends on the initial diagnosis and the progression of the disease. So far, plasma exchange with direct removal of blood lipid, having been mentioned in the category III of the American Society for Apheresis 2010 guidelines, has been wildly used for hyperlipidemia treatment. However, whether plasma exchange has a beneficial effect on severe hyperlipidemia is still in dispute. In contrast, quite a few single-center clinical studies have shown that the efficacy of conservative treatment is similar to plasma exchange in lowering the serum triglyceride level, and there is no substantial difference between the two approaches in the overall benefit, specifically in terms of morbidity and mortality (24–26). In addition, the blood lipid concentration may return to the pre-apheresis levels after plasma exchange, while low-fat formula diet collocated with pharmacological therapy may be more gentle and sustainable. Moreover, the lack of availability of plasma exchange in most of medical centers as well as its rather high costs also limits its use in clinical settings. As a result, invasive therapeutic options such as plasma exchange were not considered as the first-line treatment in this case.

During hospitalization, since the patient's TG level once raised up to 25.46 mmol/L, putting her at a high risk of pancreatitis, oral medication (low-fat diet) had been suspended and replaced by total parenteral nutrition till her condition was stable, followed by a low-fat diet in combination with pharmacological therapy. This therapeutic option was proved to be sufficient in rapidly reducing the plasma TG to a safe level, and in maintaining the serum TG and TC within a low to medium range, which was also a key factor to ensure the normal growth of the patient in the following months.

### Molecular Analysis

As is known that elevated plasma triglyceride may, as an independent risk factor, lead to cardiovascular diseases (CVDs). LPL plays a critical role in lipid metabolism and energy balancing by hydrolyzing the triglyceride in blood circulation. Although it is parenchymal cells that synthesize and secret LPL, it somehow acts on the luminal capillary endothelium, so the mechanism of LPL migrating into the luminal capillary has long been among the list of relevant research (27). According to previous studies, hyperlipidemia was defined as two categories: severe (triglyceride concentration >10 mmol/L) and mild-to-moderate (triglyceride concentration 2–10 mmol/L). The former is more likely to have a monogenic cause triggered by gene variants which consequently cause LPL dysfunction (28–30). It has been understood that multiple factors can interact with LPL, positively or negatively, thus exerting an influence on TG lipolysis. Among them is a recently identified factor GPIHBP1, which is considered indispensible in transportation of LPL to the luminal capillary endothelium as well as in the establishment of the platform for TG hydrolysis (31). Currently, a large amount of evidence supports that GPIHBP1 functions in triglyceride-rich lipoprotein (TRL) metabolism of human in unique and diverse ways (32–34). Recent studies have shown that LPL mislocalization as a result of GPIHBP1 deficiency may cause severe hyperlipidemia (13, 35). Therefore, the importance of GPIHBP1 in lipolysis is being recognized more widely with more relevant research published.

The human *GPIHBP1* gene consists of 4 exons which encode a 184 amino acid protein. An acidic domain being able to bind LPL and chylomicrons is located in the N-terminus of *GPIHBP1*. Its C-terminus is encoded by exons 3 and 4, including a cysteine-rich lymphocyte antigen 6 (Ly6) motif as well as a carboxylterminal hydrophobic sequence involved in the addition of a GPI anchor (36–39). A bunch of residues in the domain of C-terminus, such as Ser107, Thr124, and Leu135 also play a critical role in LPL binding and its transportation from subendothelial space to luminal capillary (40). Therefore, deleting exons 3 and 4 will produce a crippled protein characterized by the absence of a domain indispensible for LPL binding and translocation as well as for anchoring LPL to the cell surface. This makes sense of the phenotype in cases with severe hyperlipidemia and also explains the lack of circulating LPL and LPL activity in heparin-processed plasma. Since the initial description of hyperlipidemia resulting from the variant of *GPIHBP1* in 2007 (41), only about 50 cases have been reported worldwide (35, 42, 43). Meanwhile, the genotypic spectrum based on HGMD has recorded 47 various pathogenic variants in *GPIHBP1* that can result in severe hyperlipidemia, with most of them (33/47) identified as missense variants. Tendency of these variants toward any ethnicity or racial groups was not observed. Therefore, thorough analysis on these disease-causing variants probably linking to developmental malfunction and phenotypic changes is essential to pathogenesis clarifying and clinical treatment.

The patient in our study predominantly manifested fever, vomiting and convulsion, with unexpected discovery of severe hyperlipidemia and molecular genetic tests revealing the etiology. With the WES method, we found a novel homozygous variant, c.45_48dupGCGG(Pro17Alafs^*^22), in exons 1 of *GPIHBP1*, which, absent in all of the 100 healthy control samples, was inherited from both of her parents.

The c.45_48dupGCGG duplication variant in exon 1 may lead to the substitution of the remaining 168 C-terminal amino acid residues with 21 mutant ones, at a highly conserved position of this protein. Based on the clinical presentations, serum lipid level and bioinformatics study, an inference stood out from the potential pathogeneses that the disease of the proband was very likely to be attributed to the homozygous duplication variant, which, certainly, needs to be further verified by functional experiments in the future. Fortunately, these unexpected findings, together with timely and proper treatment, have resulted in a good prognosis observed in this patient. Furthermore, this orphan case gave us a lively lesson, and deepened our knowledge of this disease.

## Conclusions

In this study, we presented a comprehensive delineation of the first-reported Chinese infant patient with severe hyperlipidemia whose condition was rooted in a novel homozygous variant of the *GPIHBP*1 gene. Our findings reveal that routine blood biochemical tests are crucial to the diagnosis, and by molecular genetic testing, an integral part of the tools for a confirmed diagnosis, the role of genetic defects as a vital predisposing factor can be identified in severe hereditary hyperlipidemia.

## Data Availability Statement

The raw data supporting the conclusions of this article will be made available by the authors, without undue reservation.

## Ethics Statement

Written informed consent was given by the parents for the publication of all associated data and images.

## Author Contributions

XZ and YZ made a great contribution to the study design and also worked as two of the major participants in genetic analyses and manuscript drafting. SW and HO were responsible for the patient's management and data collection. JL and NC conducted the whole exome sequencing, variant verification, and bioinformatics analysis of the variants. WZ and JJ performed clinical routine biochemistry tests. SL and ZW conceived the study, at the same time worked as coordinators in this study, and assisted in manuscript drafting. All the authors have reviewed, provided comments, and approved the manuscript as the final version.

## Funding

This research was funded by Natural Science Foundation of Guangdong Province (No. 2021A1515010969) and Research Project of Traditional Chinese Medicine Bureau of Guangdong Province (No. 20211046). The sponsors were not involved in study design, data analyzing or interpreting. Neither did they contribute to developing the report.

## Conflict of Interest

The authors declare that the research was conducted in the absence of any commercial or financial relationships that could be construed as a potential conflict of interest.

## Publisher's Note

All claims expressed in this article are solely those of the authors and do not necessarily represent those of their affiliated organizations, or those of the publisher, the editors and the reviewers. Any product that may be evaluated in this article, or claim that may be made by its manufacturer, is not guaranteed or endorsed by the publisher.

## Abbreviations

TG: triglyceride
TC: total cholesterol
HDL-C: high-density lipoprotein cholesterol
LDL-C: low-density lipoprotein cholesterol
LPL: lipoprotein lipase
APOC2: apolipoprotein CII
APOA5: apolipoprotein AV
LMF1: lipase maturation factor 1
GPIHBP1: glycosylphosphatidylinositol-anchored highdensity lipoprotein-binding protein 1
Ly6: lymphocyte antigen 6
MCT: medium chain triglycerides
TPN: total parenteral nutrition
WES: whole exome sequencing
BWA: Burrows-Wheeler Aligner
GATK: Genome Analysis Toolkit
SNVs: single-nucleotide variants
OMIM: Online Mendelian Inheritance in Man
FATHMM: Functional Analysis through Hidden Markov Models
CVD: cardiovascular disease.
