# Supplementary material for: Case Report: Successful Management of a 29-Day-Old Infant With Severe Hyperlipidemia From a Novel Homozygous Variant of GPIHBP1 Gene
Source: Front Pediatr. 2022 Mar 10;10:792574. doi: 10.3389/fped.2022.792574 (PMC8960264; doi:10.3389/fped.2022.792574)
Supplement: Supplementary file 1 [file Table_1.doc]

**Supplementary table 1 Episode of the patient's treatment process in hospital**

| **Date** | **Age** | **Therapeutic Purpose** | **Therapeutic Schedule** | **Route of Administration** | **Dosage** |
| --- | --- | --- | --- | --- | --- |
| 2020-09-06 | 29d | - | deep vein puncture | - | - |
| 2020-09-06 | 29d | nutritional support | amino acid-based formulae | oral administration | 20ml/each time, q3h |
| 2020-09-07 | 30d | improve anemia | red cells suspension | venous inflow | 0.5U, one time |
| 2020-09-07 | 30d | improve hypoproteinemia | albumin | venous inflow | 4g, one time |
| 2020-09-10 | 1m2d | improve coagulation function | fresh frozen plasma  (blood type O, rhesus positive) | venous inflow | 70ml, one time |
| 2020-09-11 | 1m3d | improve hypoproteinemia | albumin | venous inflow | 4g, one time |
| 2020-09-12 | 1m4d | improve hypoproteinemia | albumin | venous inflow | 4g, one time |
| 2020-09-12 | 1m4d | anti infection | vancomycin | venous inflow | 10mg/kg, q6h, last for 9 days |
| 2020-09-12 | 1m4d | anti infection | meropenem | venous inflow | 40mg/kg, q8h, last for 9 days |
| 2020-09-12 | 1m4d | total parenteral nutrition | 10% glucose injection, 6.74% amino acid injection, 50% glucose injection, 10% NaCl and KCl injection | venous inflow | bid, last for 7 days |
| 2020-09-25 | 1m17d | nutritional support | low-lipid formula diet  (special milk powder) | oral administration | 30ml/each time, q3h |
| 2020-10-11 | 2m3d | - | discharged | - | - |
