# Supplementary material for: Case Report: Successful Management of a 29-Day-Old Infant With Severe Hyperlipidemia From a Novel Homozygous Variant of GPIHBP1 Gene
Source: Front Pediatr. 2022 Mar 10;10:792574. doi: 10.3389/fped.2022.792574 (PMC8960264; doi:10.3389/fped.2022.792574)
Supplement: Supplementary file 2 [file Table_2.doc]

**Supplementary table 2: Therapeutic doses of the drugs for the patient with severe hyperlipidemia.**

| **Drug** | **Drug-delivery** | **Initial dose (last for one month)** | **Maximum dose** | **Maintenance dose** |
| --- | --- | --- | --- | --- |
| L-carnitin^1^ | orally | 100 mg/kg/d, tid | 3000 mg/d, bid | 50 mg/kg/d, bid |
| Coenzyme Q10^2^ | orally | 10 mg/each time, tid | / | 10 mg/each time, bid |
| Vitamin B1^3^ | orally | 10 mg/each time, tid | / | 10 mg/each time, bid |
| Vitamin B2^4^ | orally | 5 mg/each time, tid | / | 5 mg/each time, bid |
| Vitamin B6^5^ | orally | 10 mg/each time, tid | / | 10 mg/each time, bid |
| Vitamin B12^6^ | orally | 15 ug/each time, tid | / | 15 ug/each time, bid |
| Vitamin C^7^ | orally | 100 mg/each time, tid | / | 100 mg/each time, bid |
| Folic acid^8^ | orally | 5 mg/each time, tid | / | 5 mg/each time, bid |

The dosage of all sorts of drugs was determined strictly following the instructions and individualized based on clinical symptoms and therapeutic effects.

1. Levocarnitine oral solution, Northeast Pharmaceutical Group Shenyang No.1 Pharmaceutical Co., Ltd., No.5 Kunming Lake Street, Shenyang Economic and Technological Development Zone, Shenyang, Liaoning, P.R.China. Each vial of the oral solution contains 1000 mg L-carnitine.
2. Coenzyme Q10 capsule, Zhejiang Hailisheng Pharmaceutical Co., Ltd., No.66 Xingang 11th Street, Xingang Park, Zhoushan Economic Development Zone, Zhoushan, Zhejiang, P.R.China. Each capsule contains 10 mg coenzyme Q10.
3. Vitamin B1 tablet, Guangdong Huanan Pharmaceutical Group Co., Ltd., Information Technology Industry Park, West Lake Industrial District, Shilong Town, Dongguan, Guangdong, P.R.China. Each tablet contains 10 mg vitamin B1.
4. Vitamin B2 tablet, Tianjin Lisheng Pharmaceutical Co.,Ltd., No.16, Saida BeiYi Road, Xiqing Economic and Technological Development Zone, Tianjin, P.R.China. Each tablet contains 5 mg vitamin B2.
5. Vitamin B6 tablet, Hangzhou Minsheng Pharmaceutical Co.,Ltd., No.36 Linping Avenue, Yuhang Economic and Technological Development Zone, Yuhang District, Hangzhou, Zhejiang, P.R.China. Each tablet contains 10 mg vitamin B6.
6. Vitamin B12 oral solution, Chengdu Dikang Technology Pharmaceutical Stock Co., Ltd., No.1 Dikang Avenue, West Gaoxin District, Chengdu, P.R.China. Each vial of oral solution (1ml) contains 3 μg vitamin B12.
7. Vitamin C tablet, Guangdong Huanan Pharmaceutical Group Co., Ltd., Information Technology Industry Park, West Lake Industrial District, Shilong Town, Dongguan, Guangdong, P.R.China. Each tablet contains 100 mg vitamin C.
8. Folic acid tablet, Fuzhou Fu Yao Pharmaceutical Co., Ltd., No. 279, Gushan Town, Jin 'an District, Fuzhou, Fujian, P.R.China. Each tablet contains 5 mg folic acid.
