# Supplementary material for: Case Report: Successful Management of a 29-Day-Old Infant With Severe Hyperlipidemia From a Novel Homozygous Variant of GPIHBP1 Gene
Source: Front Pediatr. 2022 Mar 10;10:792574. doi: 10.3389/fped.2022.792574 (PMC8960264; doi:10.3389/fped.2022.792574)
Supplement: Supplementary file 3 [file Table_3.doc]

**Supplementary table 3 Laboratory lipid profiles of the proband at follow-ups**

| **Date** | **Age** | **TG (mmol/L)** | **TC (mmol/L)** | **HDL (mmol/L)** | **LDL (mmol/L)** | **APO-A1 (g/L)** | **APO-B (g/L)** | **Glu (mmol/L)** |
| --- | --- | --- | --- | --- | --- | --- | --- | --- |
| 2020-09-06 | 29d | 25.46 | 14.53 | 1.12 | 2.01 | 0.41 | 0.82 | 1.8 |
| 2020-09-07 | 30d | 7.33 | 8.17 | 0.79 | 4.59 | - | - | 4.2 |
| 2020-09-08 | 1m | - | - | - | - | - | - | 3.8 |
| 2020-09-09 | 1m1d | - | - | - | - | - | - | 4.4 |
| 2020-09-10 | 1m2d | - | - | - | - | - | - | 4.7 |
| 2020-09-11 | 1m3d | - | - | - | - | - | - | 3.9 |
| 2020-09-13 | 1m5d | 14.36 | 11.19 | 0.98 | 6.95 | - | - | - |
| 2020-09-20 | 1m12d | 1.50 | 9.66 | 1.19 | 7.23 | 0.72 | 1.30 | - |
| 2020-09-23 | 1m15d | 1.80 | 10.30 | 1.41 | 7.56 | 0.70 | 1.42 | - |
| 2020-09-25 | 1m17d | 3.77 | 10.20 | 1.41 | 7.44 | 0.69 | 1.69 | 5.4 |
| 2020-09-30 | 1m22d | 3.76 | 8.45 | 1.11 | 6.05 | 0.70 | 1.35 | - |
| 2020-10-02 | 1m24d | 48.7 | 7.20 | 0.51 | 3.61 | 1.01 | 1.12 | 6.5 |
| 2020-10-04 | 1m26d | 2.07 | 4.33 | 0.66 | 3.05 | 0.64 | 0.88 | - |
| 2020-10-10 | 2m2d | 2.92 | 7.71 | 0.99 | 5.43 | 0.98 | 1.43 | - |
| 2020-10-22 | 2m14d | 1.79 | 3.96 | 0.95 | 2.60 | 1.02 | 0.69 | - |
| 2020-11-20 | 3m12d | 4.38 | 3.79 | 0.82 | 2.53 | 0.90 | 0.77 | - |
| 2021-01-14 | 5m6d | 4.96 | 3.85 | 0.72 | 2.54 | 0.89 | 0.94 | - |
| 2021-05-21 | 9m13d | 6.64 | 3.96 | 0.67 | 2.49 | 0.90 | 0.86 | 4.2 |

**TG:** Triglyceride; **TC:** Total cholesterol; **HDL-C:** High-density lipoprotein cholesterol; **LDL-C:** Low-density lipoprotein cholesterol; **APO-A1:** Apoprotein A1; **APO-B:** Apoprotein B; **Glu:** Glucose.
