# Supplementary material for: Case Report: Successful Management of a 29-Day-Old Infant With Severe Hyperlipidemia From a Novel Homozygous Variant of GPIHBP1 Gene
Source: Front Pediatr. 2022 Mar 10;10:792574. doi: 10.3389/fped.2022.792574 (PMC8960264; doi:10.3389/fped.2022.792574)
Supplement: Supplementary file 4 [file Data_Sheet_1.doc]

**Whole exome sequencing (WES)**

A QIAamp DNA blood mini kit (cat. no. 51104; Qiagen GmbH) was employed to extract Genomic DNA from buffy coat under the manufacturer’s instructions. Illumina’s TruSeq Exome Enrichment Kit was used to enrich and sequence the exomes of the patient and her healthy parents. Consequently, a total of 62 Mb tragets were tested in WES procedure, including 20,000 plus genes , 200,000 plus exons, and 9 Mb of predicted microRNA targets. Illumina HiSeq 2000 used to perform paired-end WES of these samples yielded 120 million reads per sample in average and averagely covered 60-80× target regions after removing the duplicates. Following validation in FastQC as a quality control, the obtained sequencing data included in the FASTQ file were processed by Burrows-Wheeler Aligner (BWA), aiming to cross-check with the human reference genome hg38. Picard was employed to remove the PCR duplicates, with a combined use of SamTools and Genome Analysis Toolkit (GATK) to screen out the single-nucleotide variants (SNVs). When the single set was established through converging the variant calls originated from both callers, ANNOVAR was applied to annotate and filter SNVs, and to identify more deleterious variants through investigating a varitey of programs and databases for each and every called position. There were multiple options in the filtering procedure in ANNOVAR: (1) A frequency threshold of >1% in the 1000 Genomes Project (1000GP) data and the Exome Sequencing Project 6500 (ESP6500) data indicates common SNVs that are less likely to have a disease-causing nature; (2) the SNVs that may be classified as the category triggering amino acid changes in the protein; (3) variants with an unclear impact on protein structure necessitating further assessment using certain prediction tools; and (4) a gene or SNV that may be associated with certain known disease by crosschecking with Online Mendelian Inheritance in Man (OMIM) or Human Gene Mutation Database (HGMD). Besides, the variants with a negative Genomic Evolutionary Rate Profiling (GERP) +++ , or with a functional analysis score >0.1 in Hidden Markov Models (FATHMM) were filtered out as well. By deducing evolutionary constraints at some specific positions in an exome, GERP can pick out the “constrained elements” where a couple of positions in combination may yield a signal as a presumptive functional element.^16^ In addition, homozygous variants from either side of the proband’s parents were also screened out.

**Frequency investigation**

The 1000 Genomes Project data (http://www.internationalgenome.org/), Exome Sequencing Project (ESP, https://evs.gs.washington.edu/EVS/), and ExAC Browser (http://exac.broadinstitute.org/) were interrogated to identify all the potentially disease-causing variants. The candidate variants, as required, had to be with an occurance of ＜1% of the population in the above databases.

**Variant verification**

Aiming to differentiate potentially pathogenic variants from those with a non-pathogenic nature, we put emphasis only on non-synonymous (NS) variants as well as splice acceptor and donor site variants (SS) with the addition of short coding indels, expecting synonymous variants unlikely to be pathogenic. The variants were then filtered by cross-checking with the public databases—Human Gene Mutation Database (HGMD professional, http://www.hgmd.cf.ac.uk/ac/index.php), ClinVar (http://www.ncbi.nlm.nih.gov/clinvar/), Human Genome variant Society (HGVS) Website (http://www.hgvs.org/dblist/glsdb.html), Catalogue of Somatic Mutations in Cancer (COSMIC, <https://cancer.sanger.ac.uk/cosmic/),> Database of Genomic variants (DGV, <http://dgv.tcag.ca/dgv/app/home)>, Genome Aggregation Database (gnomAD, <http://gnomad.broadinstitute.org/),> 1000 Genomes Project (http://www.internationalgenome.org/), and Leiden Open variant Database (LOVD, http://www.lovd.nl/3.0/home). Any variant absent from these datasets was defined as novelty. Only recessive inheritance, that is, autosomal recessive model and X-linked recessive model, was taken into consideration in light of the normal phenotypes of the proband’s parents. Validation of the WES results was performed by Sanger sequencing, which was also employed to distinguish between true variants and sequencing artifacts for the spotted potentially disease-causing variants. The pathogenicity of the variants was evaluated by in silico prediction algorithms-Mutation Taster, PROVEAN, and CADD. To verify the novelty of the variants found in the present study instead of an SNP, one hundred Chinese-originated healthy subjects with no blood lineage or a hyperlipidemia family history were also enrolled in the study as control.

**Assessment of the conservation of amino acid residues**

Homologous proteins originated from eight eukaryotic species were employed for alignment with the amino acid sequences of the target human proteins in this study, including Pan troglodytes (chimpanzee), Macaca mulatta (Rhesus monkey), Loxodonta africana (African savanna elephant), Ovis aries (sheep), Felis catus (domestic cat), Rattus norvegicus (Norway rat), Mus musculus (house mouse), and Panthera pardus (leopard). UCSC Genome Browser (<http://genome.ucsc.edu/>) was used to collect the amino acid sequences. Functional and structural variations responsible for these amino acid changes were predicted by PolyPhen-2 (http://genetics.bwh.harvard.edu/pph2/), Sorting Tolerant From Intolerant (SIFT, https://sift.bii.a-star.edu.sg/), and MutationTaster (http://www.mutationtaster.org/).
